# Improving cardiometabolic screening on an inpatient psychiatric ward: a quality improvement project

**DOI:** 10.1192/bjo.2021.522

**Published:** 2021-06-18

**Authors:** Harrison Howarth, Jonathan Pass, Fahel Ahmed, Sarah Wiethoff

**Affiliations:** Camden and Islington Foundation NHS Trust

## Abstract

**Aims:**

Primary aim: To increase the proportion of patients receiving a full cardiometabolic screen whilst on the ward to 75%.

Secondary aims: To improve communication with GPs regarding cardiometabolic health, to improve the rates of intervention when abnormalities are found to 75%.

**Background:**

People with serious mental illness are known to have significantly increased risk of cardiometabolic syndrome than the general population. Estimates suggest there would be up to 12,000 fewer deaths from cardiovascular disease if people with serious mental illness had the same outcomes as the general population. People with serious mental illness die on average 20 years earlier than the general population due to preventable physical health problems.

Whilst on the ward, we have an excellent opportunity to screen and treat patients with cardiometabolic risk factors, yet screens are often incomplete, not acted upon, or simply not carried out.

**Method:**

Using the Plan-Do-Study-Act (PDSA) methodology, we trialed interventions to improve the cardiometabolic screening process on out 16 bed inpatient ward. Across 8 cycles, we set up a protocol to ensure all new patients received a full cardiometabolic screen during their admission reviews, engaged nursing staff with the process and managed inconsistencies with blood transportation and delivery. We also started using British Heart Foundation information leaflets, and treating patients in accordance with the Lester Tool: Positive Cardiometabolic Health Resource. We made design changes to the discharge summary template allowing for clear communication with GPs on discharge.

**Result:**

At the end of 8 cycles, we had achieved 100% compliance with the full cardiometabolic screen (as defined by the Lester Tool) from a baseline of just 25%. We also improved intervention with identified abnormalities from a baseline of 0% to 100%.

**Conclusion:**

Improvements in cardiometabolic screening and treatment were possible using the PDSA methodology. Given the success of this quality improvement project, we plan to introduce our methodology onto other wards in the trust.

